# Serum uric acid predicts therapeutic response to midodrine hydrochloride in children with vasovagal syncope: a pilot study

**DOI:** 10.1007/s00431-023-05297-2

**Published:** 2023-10-31

**Authors:** Xiaojuan Du, Xueying Li, Chunyu Zhang, Ping Liu, Yuli Wang, Qingyou Zhang, Junbao Du, Ying Liao, Hongfang Jin

**Affiliations:** 1 Department of Pediatrics, Peking University First Hospital, Beijing, 100034 China; 2 Department of Statistics, Peking University First Hospital, Beijing, 100034 China; 3 State Key Laboratory of Vascular Homeostasis and Remodeling, Peking University, Beijing, 100191 China

**Keywords:** Uric acid, Therapeutic effect, Midodrine hydrochloride, Vasovagal syncope, Children

## Abstract

Serum uric acid (UA) level has been proven to be related to several cardiovascular and metabolic diseases. In the present study, we examined if baseline serum UA level could predict the therapeutic efficacy of midodrine hydrochloride on vasovagal syncope (VVS) in children. The pediatric VVS patients who received midodrine hydrochloride from November 2008 to October 2022 were enrolled. After a median treatment duration of 3 months, the therapeutic effect was evaluated. According to the patients’ responses to midodrine hydrochloride, which was determined by the recurrence of syncope, they were divided into effective and ineffective groups. The baseline variables were explored using univariable and multivariate logistic analysis. The predictive efficacy was assessed by receiver operating characteristic curve (ROC), precision-recall curve (PR), Hosmer–Lemeshow test, calibration curve, and decision curve analysis (DCA). Totally, 53 participants were included in the study. Among the 51 patients who were successfully followed up, 29 (56.9%) responded to midodrine hydrochloride (effective group), and the other 22 (43.1%) failed to respond to midodrine hydrochloride (ineffective group). The participants in effective group had lower baseline serum UA level than those in ineffective group (276.5 ± 73 μmol/L vs. 332.7 ± 56 μmol/L, *p* = 0.004). Multivariable logistic analysis showed that serum UA was associated with the therapeutic response (odds ratio (OR): 0.985, 95% confidence interval (CI): 0.974–0.997, *p* = 0.01). ROC analysis indicated that using baseline serum UA < 299 μmol/L as a threshold value yielded a sensitivity of 77.3% and a specificity of 79.3% in predicting the treatment response to midodrine hydrochloride. The area under the PR curve was 0.833. Hosmer–Lemeshow test yielded a *p* value of 0.58, and calibration plot indicated that the model was well-fitted. DCA demonstrated that treatment decision depending on the baseline serum UA level resulted in a favorable net benefit.

*Conclusion*: This pilot study suggested that the baseline serum UA level could be taken as a predictor of therapeutic effect of midodrine hydrochloride on VVS in children.

**Table:** 

**What is Known:** *• Empirical and unselected use of midodrine hydrochloride has an unfavorable therapeutic effect on VVS in children. Serum uric acid (UA) is closely linked to cardiovascular events.*	
**What is New:** *• A low baseline serum UA level successfully predicts the therapeutic effectiveness of midodrine hydrochloride on VVS in children.*

**What is Known:**

*• Empirical and unselected use of midodrine hydrochloride has an unfavorable therapeutic effect on VVS in children. Serum uric acid (UA) is closely linked to cardiovascular events.*

**What is New:**

*• A low baseline serum UA level successfully predicts the therapeutic effectiveness of midodrine hydrochloride on VVS in children.*

## Introduction

Typically, the onset of vasovagal syncope (VVS) begins in adolescence age [1], and the pathogenesis is complex [2]. Although most cases of VVS have a favorable prognosis, up to one-third of those affected will experience recurrent attacks and have a risk of physical trauma as well as mental problems [3]. It is difficult and challenging to treat pediatric VVS cases due to the multiple pathogenesis of this syndrome, including vascular dysfunction, hypovolemia, and paradoxical reflexes [4, 5]. Midodrine hydrochloride is an alpha1-adrenergic receptor agonist and a vasoconstrictor that can avoid the blood stasis in peripheral vascular beds. It may consequently prevent the drop in cardiac output caused by a decreased preload due to the blood redistribution during standing, and then inhibits a vasovagal response [6]. Although midodrine hydrochloride is an effective treatment in early random control trial [7], 40% of those treated with midodrine hydrochloride experienced a syncope or presyncope recurrence [8] likely due to the diversity of the pathogenesis of VVS. Considering this, it is crucial to identify the baseline indicators that might predict the patients’ responses to midodrine hydrochloride, hence enhancing its therapeutic efficacy.

Hyperuricemia, defined as serum uric acid (UA) levels exceeding 420 μmol/L for males and 360 μmol/L for females [9], is considered to be closely linked to cardiovascular events [10, 11]. A recent study proved the serum UA level in distinguishing between generalized tonic–clonic seizures, syncope, and psychogenic nonepileptic seizures [12]. In addition, severe hypouricemia impaired endothelium-dependent vasodilation and lowered blood pressure in young adults [13]. Since abnormal endothelium-dependent vasodilation was also found in children with neurally mediated syncope including VVS [14, 15], we intended to examine whether baseline serum UA level in children with VVS could be a possible predictor of the effectiveness of midodrine hydrochloride.

## Methods

### Study design and patients

This study consisted of 53 children with VVS. After follow-up, 51 entered the analysis, including 20 males and 31 females, at a median age of 13.0 (11.0, 14.0) years old.

Diagnostic criteria for VVS [16]: (1) syncopal episodes are frequently triggered by factors that render individuals more susceptible, including changes in posture from supine to standing up, prolonged periods of upright posture, and exposure to hot and humid environments; (2) experiencing episodes of syncope; (3) with a positive response during a head-up tilt test (HUTT); and (4) ruling out alternative causes of syncope-like events, such as epilepsy, hypoglycemia, and cardiac syncope.

Inclusion criteria: the children patients (1) diagnosed with VVS at the pediatric syncope unit of Peking University First Hospital from November 2008 to October 2022 and (2) treated with midodrine hydrochloride.

Exclusion criteria: the children patients (1) with no syncopal attacks within 3 months before their admission; (2) had other reasons for transient loss of consciousness (such as cardiogenic syncope, epilepsy, or psychogenic pseudosyncope); and (3) received other drugs in the treatment (such as metoprolol and pyridostigmine) at the same time.

The study was approved by the Institutional Ethics Committees of Peking University First Hospital (No. 2022496), and informed consent was obtained from individual participants and their parents.

### Data collection and detection of variables

Demographic information, symptoms, and baseline hemodynamic parameters were obtained by searching electronic medical records (Donghua, Beijing, China and Kaihua, Beijing, China). Baseline serum biochemical variables (serum UA, glucose, triglyceride, total cholesterol, phosphorus, and magnesium) were detected.

Venous blood was collected after fasting 12 h. Serum UA level was measured by uricase colorimetric method. Blood glucose level was analyzed by glucose oxidase method. Serum total cholesterol and triglyceride levels were analyzed by enzymatic method. Serum magnesium and phosphorus levels were detected by colorimetric method.

### Head-up tilt test (HUTT)

HUTT [16] was done in an environment with low lighting, warm temperature, and minimal ambient noise as the previous description. Prior to undergoing HUTT, patients were required a fasting period of at least four hours. Additionally, it was necessary for patients to discontinue the use of medications that could impact the autonomic nervous system for a duration equivalent to five half-lives of the respective medication. The participants were observed for a duration of 20 min while in a supine position on a tilt table (SHUT-100A, Standard, Jiangsu, and ST-711, Juchi, Beijing, China). The tilt table was inclined at an angle of 60 degrees, and the test was continued until either a positive reaction occurred, or a duration of 45 min elapsed. Positive hemodynamic responses are determined as follows: (1) significant hypotension (systolic blood pressure ≤ 80 mmHg, diastolic blood pressure ≤ 50 mmHg, or a ≥ 25% decrease in mean blood pressure), (2) bradycardia (heart rate < 75 bpm in children aged 4–6 year, < 65 bpm in those aged 6–8 years, and < 60 bpm in those aged > 8 years), (3) sinus arrest, or (4) second-degree or greater atrioventricular block and asystole persisting for > 3 s [17]. There are correspondingly three hemodynamic types named as vasodepressor, cardioinhibitory, and mixed type. The vasodepressor response is characterized by a marked decrease in blood pressure without an obvious reduction in heart rate. The cardioinhibitory response, on the other hand, is characterized by a noticeable decrease in heart rate without a significant decrease in blood pressure. Lastly, the mixed response involves both a noticeable decrease in blood pressure and a significant reduction in heart rate.

### Treatment protocol and follow-up

The patients were treated with midodrine hydrochloride at a dosage of 1.25 mg twice daily [17]. Median treatment duration was 3.0 (2.0, 3.0) months. After treatment, the therapeutic effect was followed up by one trained researcher over telephone or clinic visits, and the main contents included the recurrence of syncope as well as the adverse effect of the drug. Two patients (3.8%) were lost from follow-up. An effective response was defined when there was no recurrence of syncope during the follow-up [17, 18].

### Statistical analysis

The baseline information was described as the mean ± standard deviation or median (interquartile range). Kolmogorov–Smirnov test was carried out for normal distributivity. The *t* test or Mann–Whitney *U* test was utilized for continuous items, and Chi-square test was utilized for categorical items. Missing data was managed using mean imputation. Multivariable logistic regression analysis (forward stepwise) was applied to select variables associated with therapeutic effect. Predictive performance was estimated by receiver operating characteristic (ROC) curve, precision-recall (PR, modEvA package) curve, calibration plot (MASS package), Hosmer–Lemeshow test, and decision curve analysis (DCA, rmda package). The optimum cut-off value was set to optimize the Youden index. The level of statistical significance was set below 0.05. For statistical analysis, we employed SPSS 26.0 (IBM Corp., NY, USA) and R 4.2.0.

## Results

### Patient characteristics

**Fig. 1:**
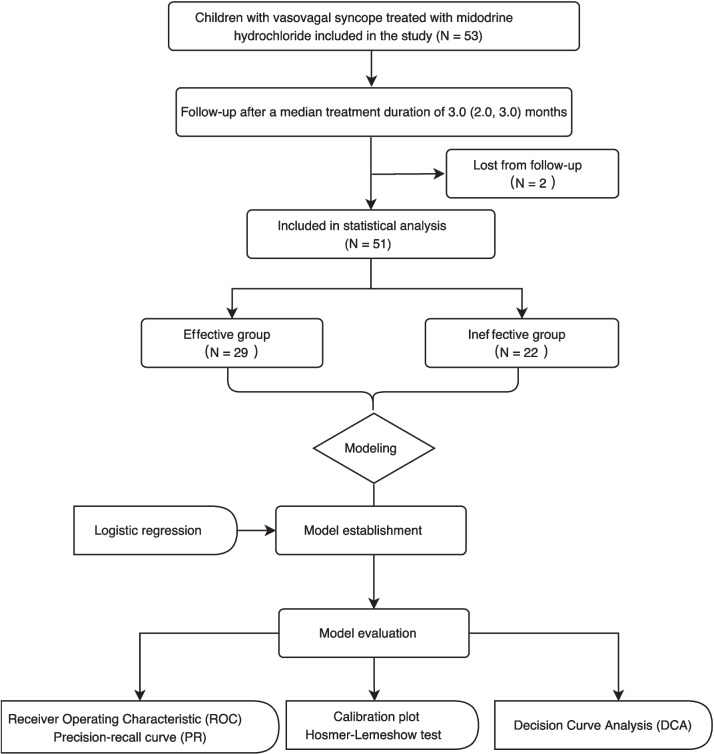
The flowchart for participant recruitment, model establishment, and evaluation in the study

**Fig. 2:**
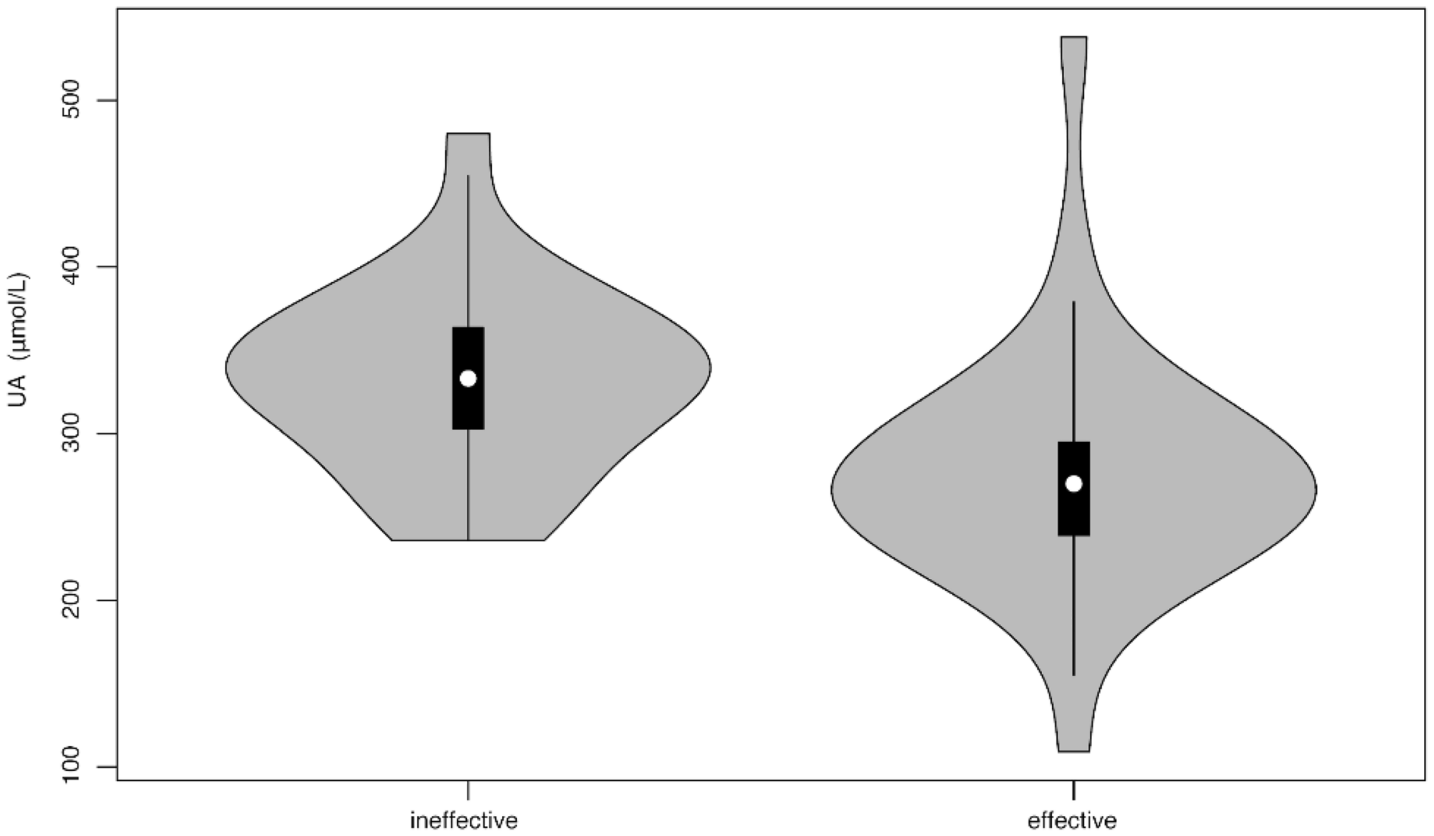
Serum UA level in midodrine hydrochloride-effective and -ineffective children with vasovagal syncope. The ordinate is the uric acid concentration. The abscissa is two groups. The level of serum UA was significantly lower in effective group than those in ineffective group (*p* = 0.004). UA: uric acid

Table 1 summarizes the baseline data of the participants (Fig. 1). About 40% of participants were male. The median age of participants was 13.0 (11.0, 14.0) years old. Among the 51 participants who had complete follow-up data, 29 cases (56.9%) responded to midodrine hydrochloride therapy (effective group), while the other 22 cases (43.1%) did not respond (ineffective group). There was no statistical difference in hemodynamic types of response in HUTT between the two groups. Participants in effective group had a shorter duration of hospital stay than those in ineffective group (7 (6, 8) days vs. 8 (7, 11) days; *p* = 0.031). The level of serum UA was significantly lower in effective group than that in ineffective group (276.5 ± 73 μmol/L vs. 332.7 ± 56 μmol/L; *p* = 0.004) (Fig. 2). The variables with incomplete data including glucose (5 missing), triglyceride (4 missing), and total cholesterol (4 missing) were calculated using mean imputation method (Table 1). No statistical differences in other characteristics existed between the two groups.

**Table 1:** Comparison of baseline characteristics between midodrine hydrochloride-effective and -ineffective children with vasovagal syncope

Items	All (*n* = 51)	Treatment response groups		*p* value
		Effective (*n* = 29)	Ineffective (*n* = 22)	
Sex (male/female, *n*)	20/31	10/19	10/12	0.427 ^a^
Age (years)	13.0 (11.0, 14.0)	13.0 (11.5, 14.0)	12.0 (10.8, 14.3)	0.624 ^b^
Body mass index (kg/m^2^)	18.5 (17.2, 20.8)	18.8 (17.2, 20.9)	18.2 (17.1, 20.5)	0.718 ^b^
Duration of treatment (months)	3.0 (2.0, 3.0)	3.0 (2.0, 3.0)	2.8 (1.9, 3.0)	0.355 ^b^
Couse of disease (months)	24.0 (5.0, 36.0)	24.0 (4.5, 71.0)	13.5 (4.8, 31.5)	0.317 ^b^
Duration of hospitalization (days)	7 (7, 10)	7 (6, 8)	8 (7, 11)	0.031 ^b^
Total attacks of syncope before treatment (times)	4 (3, 6)	4 (2, 7)	4 (3, 6)	0.759 ^b^
Response duration in HUTT (minutes)	13 (5, 26)	16 (5, 25)	13 (6, 28)	0.668 ^b^
Hemodynamic types of response in HUTT				0.123 ^a^
Vasodepressor	40	20	20	
Cardioinhibitory + mixed	11	9	2	
Heart rate (bpm)	74 (68, 83)	75 (68, 81)	71 (66, 85)	0.827 ^b^
Systolic blood pressure (mmHg)	109 ± 12	110 ± 11	108 ± 14	0.588 ^a^
Diastolic blood pressure (mmHg)	64 ± 78	64 ± 76	63 ± 79	0.471 ^c^
Serum uric acid (μmol/L)	300.8 ± 71	276.5 ± 73	332.7 ± 56	0.004 ^c^
Serum glucose (mmol/L)	4.7 (4.5, 5.0)	4.7 (4.6, 5.0)	4.8 (4.5, 5.0)	0.917 ^b^
Serum triglyceride (mmol/L)	0.9 (0.7, 1.1)	0.9 (0.7, 1.1)	0.9 (0.8, 1.1)	0.397 ^b^
Serum total cholesterol (mmol/L)	3.7 ± 0.6	3.7 ± 0.7	3.8 ± 0.5	0.700 ^c^
Serum phosphorus (mmol/L)	1.5 ± 0.2	1.5 ± 0.2	1.6 ± 0.2	0.403 ^c^
Serum magnesium (mmol/L)	0.85 ± 0.07	0.86 ± 0.06	0.85 ± 0.07	0.842 ^c^

^a^*p* value of chi-square test; ^b^*p* value of Mann–Whitney *U* test; ^c^*p* value of t test

Glucose: 5 missing, triglyceride: 4 missing, total cholesterol: 4 missing

*HUTT *head-up tilt test, *bpm* beat per minute

### Multivariate logistic regression

We used multivariate logistic regression to analyze variables associated with therapeutic effect. Six factors (age, body mass index, sex, duration of hospitalization, course of disease, and serum UA) were included to construct a multivariate logistic regression. The results demonstrated that serum UA was an independently associated variable with therapeutic effect (odds ratio (OR) = 0.985, 95% confidence interval (CI) 0.974–0.997, *p* = 0.011).

### Performance evaluation metric

**Fig. 3:**
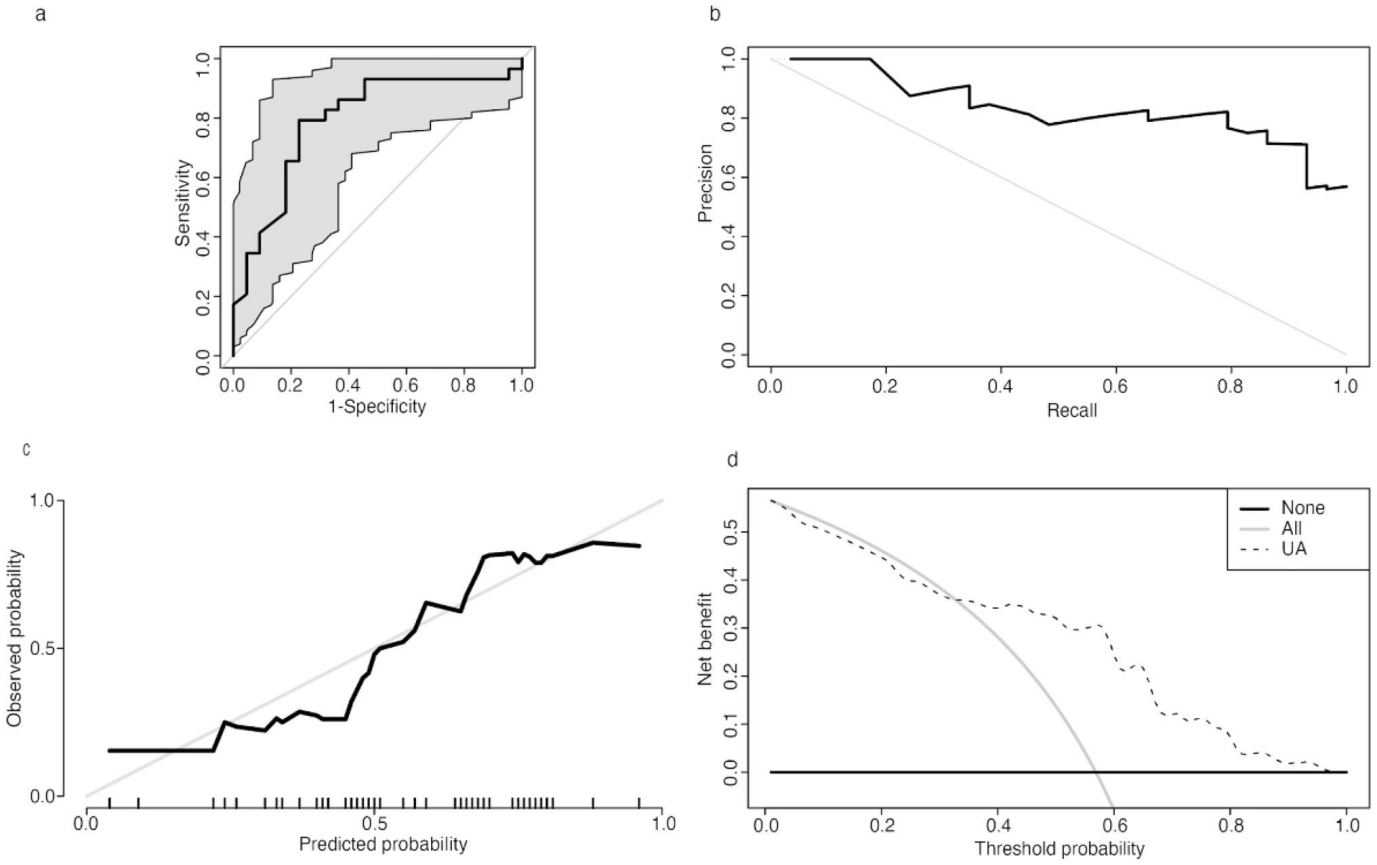
Prediction model for evaluating effectiveness of midodrine hydrochloride in children with vasovagal was assessed by receiver operating characteristic (ROC) curve, precision-recall (PR) curve, calibration curve, and decision curve analysis (DCA). **a** ROC curve. Specificity (the abscissa): 79.3%. Sensitivity (the ordinate): 77.3%. Area under the curve was 0.79 with a 95% confidence interval of 0.66–0.92. **b** PR curve. Precision is the abscissa and recall is the ordinate. Area under the curve was 0.833. **c** Calibration curve. The abscissa is the predicted incidence of events (predicted probability), and the ordinate is the observed incidence of actual events (observed probability). The gray line on the diagonal indicates that the predicted and actual probabilities of the present model are identical. The black line represents the actual predictive accuracy of the model. The calibration curve is well-fitted. **d** DCA. *Y* axis equals the net benefit, and *x* axis equals the threshold probability. Serum uric acid-based treatment decision resulted in greater net benefit over that without uric acid especially when threshold probability > 0.35

To study the predictive ability, we conducted ROC and PR analysis. The result of the ROC curve showed an area under curve (AUC) was 0.79 (95% CI 0.66–0.92). The appropriate cut-off value of 299 μmol/L yielded a sensitivity of 77.3% and a specificity of 79.3%, respectively, to predict the effectiveness of midodrine hydrochloride on pediatric VVS (Fig. 3a). PR curve showed that the AUC was 0.833 (Fig. 3b). For the calibration, we conducted Hosmer–Lemeshow test and calibration curve. Hosmer–Lemeshow test showed that *p* was 0.58, indicating that the predicted and actual values did not significantly differ. Calibration plots revealed a good predictive accuracy (Fig. 3c). For the clinical utility, we conducted DCA curve. It demonstrated that the net benefit of serum UA varied between 0.01 and 0.54 when the threshold probability was between 0.1 and 1.0 (Fig. 3d), indicating that serum UA-based treatment decisions would result in a greater net benefit over that without UA when threshold probability was set > 0.35.

## Discussion

In this pilot study, we examined the serum UA level and examined if it could predict the therapeutic effectiveness of midodrine hydrochloride in children with VVS. We revealed that the baseline serum UA level was significantly lower in the midodrine hydrochloride-effective group than that in the ineffective group. Moreover, the serum UA level was associated with the efficacy of midodrine hydrochloride in the treatment of VVS in children. Furthermore, the children suffering from VVS with baseline serum UA < 299 μmol/L were more likely to respond to midodrine hydrochloride.

Midodrine hydrochloride was utilized for VVS children in multiple studies [7, 19–21]. However, the effectiveness of unselected use of midodrine hydrochloride on VVS in children varied. The reason of the diverse efficacy might be due to the complex pathogenesis of VVS [8], including the excessive vasodilation, hypovolemia, and paradoxical reflex. Midodrine hydrochloride can antagonize the excessive vasodilation, increase peripheral vascular resistance, and decrease the pooling of blood in the vascular bed of abdominal cavity and lower extremities. Therefore, it is necessary to find out some biomarkers indicating the pathogenesis of vascular dysfunction to help implement midodrine hydrochloride for VVS in children.

In one study, young adults with lower UA level have impaired heat-induced endothelium-dependent microvascular vasodilation [13]. Excessive UA level decreased nitric oxide bioavailability through elevating asymmetric dimethylarginine levels [22]. It was reported that flow-mediated vasodilation (FMD) correlated inversely with UA level [23]. Our study found that serum UA level in the effective group was reduced, suggesting that children with lower UA are more likely to suffer from excessive vasodilation.

Furthermore, we explored the ROC curve to evaluate the predictive ability of baseline serum UA level to the therapeutic effect of midodrine hydrochloride on the VVS in children. The results showed that using baseline serum UA < 299 μmol/L as a threshold value yielded a sensitivity of 77.3% and a specificity of 79.3% in predicting the treatment response to midodrine hydrochloride. Subsequently, an AUC of PR curve was 0.833, indicating that our proposed predictive measure has achieved a good performance. The results obtained from Hosmer–Lemeshow test and calibration plot also supported its prediction accuracy. In contrast to traditional biostatistical methods, which only evaluate the accuracy of a model, DCA showed us whether using a model for clinical decision-making would improve patient outcomes [24]. In our study, the DCA curves of the model acquired a relatively broad range of net benefit between 0.01 and 0.54, indicating that the model-based intervention in clinical practice might benefit patients.

In 2012, it was discovered that FMD could predict the effectiveness of midodrine hydrochloride but only 24 patients participated in that trial [25]. Moreover, a professionally trained and experienced technician was required to detect FMD, which limited the wide use in the clinical practice. In 2022, Li et al. found that calcitonin gene-related peptide could be another indicator of the midodrine hydrochloride therapy with a predictive sensitivity of 97.7% and a specificity of 83.3% [26]. Despite of high predictive value, clinical researchers have been looking for analogs due to the short half-life (< 30 min) of that peptide and peptide properties [27]. The peptide circulates in the plasma with a circadian rhythm [28], which makes it less suitable to be a biomarker. Serum UA is affordable and easy to detect. Therefore, serum UA level has promising clinical application for predicting the efficacy of midodrine hydrochloride on VVS in children.

Obviously, there were some limitations in the research. First, this was a retrospective study with relatively small sample size and the duration of follow-up was not long enough. Second, the dosage of midodrine used in our study is the minimum effective dose referring to the guideline [16]. It is necessary to confirm the results in patients using midodrine with higher-dose and longer-course. We also agree that perspective, multi-center, and large-sample studies should be done in the future to confirm this conclusion.

Anyway, our study revealed that serum UA level might act as a biomarker with an acceptable specificity and sensitivity for predicting the therapeutic effect of midodrine hydrochloride on VVS in children. Further study can be considered to explore the relationship between UA levels and mechanisms of VVS.

## Data Availability

The dataset analyzed during the current study are available from the corresponding author upon reasonable request.

## Abbreviations

AUC: Area under curve
CI: Confidence interval
DCA: Decision curve analysis
FMD: Flow-mediated vasodilation
HUTT: Head-up tilt test
OR: Odds ratio
PR: Precision-recall
ROC: Receiver operating characteristic
UA: Uric acid
VVS: Vasovagal syncope
